# The broad-spectrum anti-DNA virus agent cidofovir inhibits lung metastasis of virus-independent, FGF2-driven tumors

**DOI:** 10.18632/oncotarget.3079

**Published:** 2014-12-31

**Authors:** Sandra Liekens, Sam Noppen, Sofie Gijsbers, Rebecca Sienaert, Roberto Ronca, Chiara Tobia, Marco Presta

**Affiliations:** ^1^ KU Leuven, Rega Institute for Medical Research, Leuven, Belgium; ^2^ Department of Molecular and Translational Medicine, University of Brescia, Brescia, Italy

**Keywords:** basic fibroblast growth factor, melanoma, metastasis, nucleoside phosphonate, survival

## Abstract

The FDA-approved anti-DNA virus agent cidofovir (CDV) is being evaluated in phase II/III clinical trials for the treatment of human papillomavirus (HPV)-associated tumors. However, previous observations had shown that CDV also inhibits the growth of vascular tumors induced by fibroblast growth factor-2 (FGF2)-transformed FGF2-*T*-MAE cells. Here, we demonstrate that CDV inhibits metastasis induced by FGF2-driven, virus-independent tumor cells. Pre-treatment of luciferase-expressing FGF2-*T*-MAE cells with CDV reduced single cell survival and anchorage-independent growth *in vitro* and lung metastasis formation upon intravenous inoculation into SCID mice. This occurred in the absence of any effect on homing of FGF2-*T*-MAE cells to the lungs and on the growth of subconfluent cell cultures or subcutaneous tumors in mice. Accordingly, CDV protected against lung metastasis when given systemically after tumor cell injection. Lung metastases in CDV-treated mice showed reduced Ki67 expression and increased nuclear accumulation of p53, indicating that CDV inhibits metastasis by affecting single cell survival properties. The anti-metastatic potential of CDV was confirmed on B16-F10 melanoma cells, both in zebrafish embryos and mice. These findings suggest that CDV may have therapeutic potential as an anti-metastatic agent and warrants further study to select those tumor types that are most likely to benefit from CDV therapy.

## INTRODUCTION

The nucleotide analogue cidofovir [(*S*)-1-(3-hydroxy-2-phosphonyl-methoxypropyl)cytosine, CDV] is a broad-spectrum antiviral agent approved (Vistide®) for the treatment of cytomegalovirus-induced retinitis in AIDS patients [1,2].

Besides its antiviral activity, CDV possesses potent anti-tumor activity in various experimental models of virus-associated tumors [3-8]. *In vitro*, CDV inhibits the proliferation of HPV-positive (HPV+) cervical carcinoma cells by accumulation of cells in the S-phase, induction of apoptosis and stimulation of the tumor suppressor proteins p53 and pRb [8,9]. Moreover, a synergistic effect of CDV and radiation was observed in HPV18+ cervical carcinoma cells [10] and in head and neck squamous cell carcinoma cells [11]. This effect was attributed, at least in part, to inhibition of angiogenesis mediated by p53-dependent reduction in vascular endothelial growth factor (VEGF) expression [10].

In keeping with these observations, CDV proved active in patients with recurrent Epstein-Barr virus-induced nasopharyngeal carcinoma (NPC) and is increasingly being used off-label to treat a variety of human papillomavirus (HPV)-induced premalignant and malignant lesions [12-15]. In fact, intralesional CVD has been one of the mainstays of adjuvant therapy in patients with recurrent respiratory papillomatosis (RRP) since 1998 [16]. Moreover, CDV has recently been evaluated in phase II clinical trials for HPV-associated high grade cervical and vulvar carcinoma and showed promise for topical treatment of cervical intraepithelial neoplasia grade (CIN) 2+ lesions [17].

Besides its virus-related antineoplastic activity, increasing evidence indicates that CDV may show therapeutic efficacy in cancer beyond the setting of viral infection. Indeed, CDV inhibits intracranial glioblastoma growth by promoting DNA double-strand breaks and apoptosis following its incorporation into the DNA [18]. Also, CDV proved effective in the treatment of patients with basal cell carcinoma [19] and cutaneous squamous cell carcinoma [20].

Fibroblast growth factor-2 (FGF2) was one of the first angiogenic factors to be described [21]. Over the years the FGF2/FGF receptor (FGFR) system has been shown to play a major role in angiogenesis, inflammation and in the development and progression of angiogenic diseases and cancer [22-24]. Moreover, accumulating evidence indicates that FGF2 is involved in resistance of tumor vascularization against VEGF inhibitor treatment. Therefore, FGF2 and FGFRs have gained interest as promising targets for drug development in cancer therapy [25,26]. In this setting, we generated a stable oncogenic FGF2-overexpressing endothelial (FGF2-*T*-MAE) cell line able to induce highly vascularized tumors that histologically resemble Kaposi's sarcoma when grafted in immunocompromised mice [4,27,28]. Thus, FGF2-*T*-MAE cells represent an interesting model of FGF2-driven tumor development and for assessing the efficacy of anti-FGF2 therapeutic approaches. Notably, CDV inhibits the growth of primary tumors induced by FGF2-*T*-MAE cells that do not express viral oncogenes. The cytostatic and pro-apoptotic activity of CDV in these cells was found to be mediated by increased p53 protein levels and inhibition of FGF2 expression [29]. Relevant to this point, CDV also inhibits the *in vivo* growth of murine B16 melanoma cells [30], a FGF2-dependent tumor cell line [26 and references therein]. Accordingly, intralesional injection of CDV has been shown to cause the regression of a cutaneous metastasis in a melanoma patient [31].

Metastasis remains the primary cause of mortality in cancer patients. This is mainly due to the fact that micrometastases largely remain unidentified. Thus, the identification and characterization of compounds able to prevent the development of micrometastases and/or metastatic colonization, remains a challenge [32,33]. Previous observations had shown that CDV exerts an anti-metastatic activity in one experimental model involving HPV+ cells [34]. In the present study, we show that CDV inhibits the metastatic growth of virus-independent, FGF2-driven FGF2-*T*-MAE and melanoma B16 tumors. Evidence is provided that this is associated with the inhibition of single cell survival properties of tumor cells.

## RESULTS

### CDV reduces single cell survival of F2T-luc2.9 cells

We previously showed that the cytostatic activity of CDV in FGF2-*T*-MAE cells increases with decreasing seeding density [29]. Therefore, we assessed whether CDV affects the capacity of luciferase-expressing FGF2-*T*-MAE cells (F2T-luc2.9 cells) to survive as single cells.

**Figure 1:**
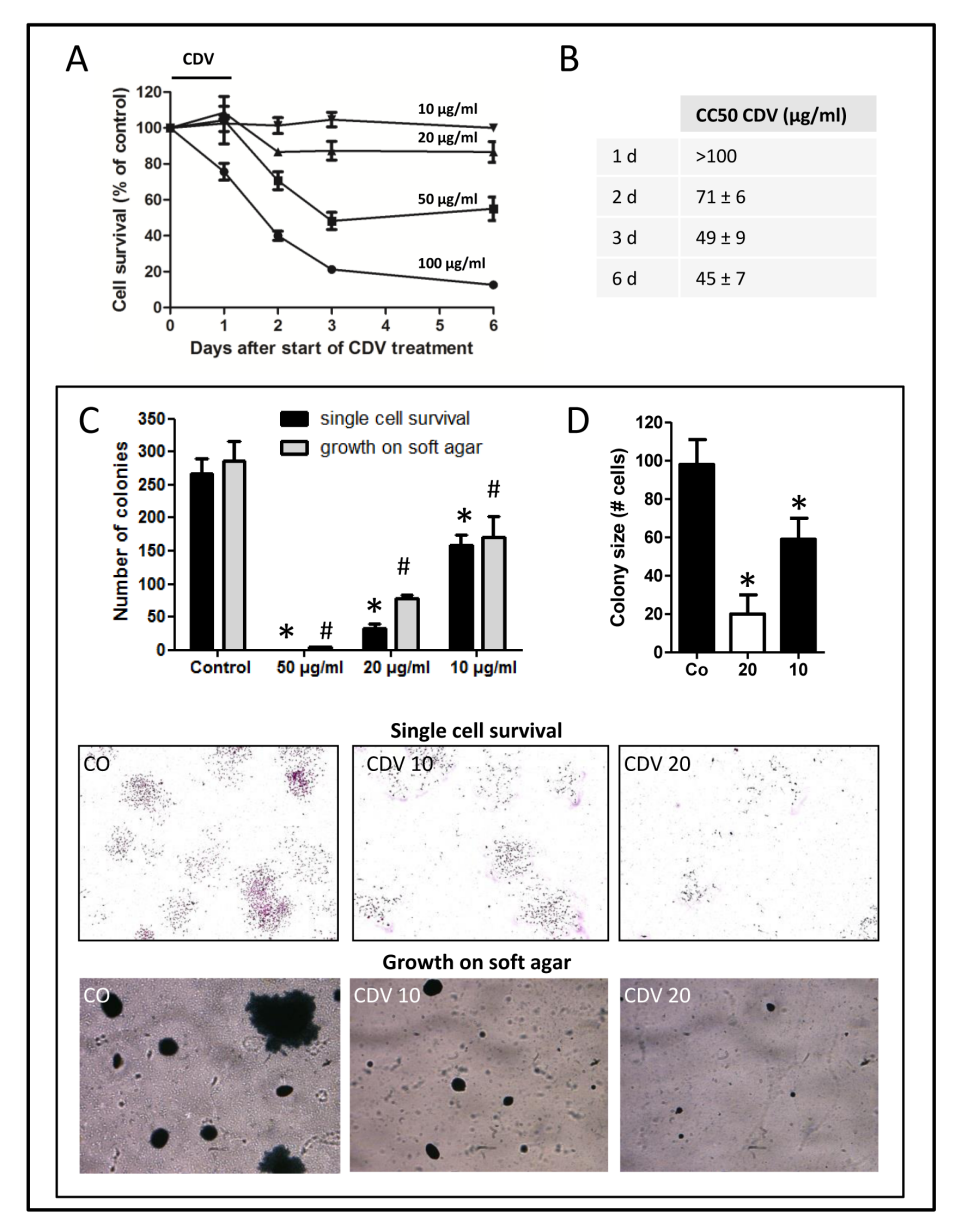
CDV inhibits single cell survival and anchorage-independent growth of F2T-luc2.9 cells F2T-luc2.9 cells were seeded at 10,000 cells/cm^2^. After overnight incubation, the cells were treated with different concentrations of CDV for 24 h. A, B, Cells were washed and incubated in fresh medium without CDV. Cell survival was measured at multiple time points by MTS assay (A) and CC_50_ values (concentration that reduces cell number by 50%) were determined (B). C, D, For single cell survival 300 pretreated cells were seeded in 6-well plates in the absence of CDV. After 7 days, the number of colonies (C) and the cells in each colony (D) were determined. *p<0.05; Student-t-test. For anchorage-independent growth 50,000 pretreated cells were seeded on soft agar in the absence of CDV. After 14 days, the colonies were counted (C). #p<0.05; Student-t-test. Representative pictures of cell colonies on culture dish (upper panel) and agar (bottom panel) are shown. Values are expressed as mean ± S.E.M. of 3 independent experiments in duplicate.

We first examined toxicity in subconfluent F2T-luc2.9 cell cultures. Cells were incubated with various concentrations of CDV for 24 h, washed and grown in fresh medium without compound for 5 days. Cell viability was determined at different time points. As previously shown for HPV+ cells (and due to the long half-life of CDV), cytotoxicity of CDV increased with time, in particular from 1 to 3 days after CDV treatment (Fig. 1A), resulting in CC_50_ values (concentration that reduces the number of living cells by 50%) that decreased from >100 μg/ml 24 h after CDV treatment to 71, 49 and 45 μg/ml after 2, 3 and 6 days, respectively (Fig. 1B).

Next, F2T-luc2.9 cells, pretreated for 24 h with different concentrations of CDV, were seeded as single cells in the absence of CDV. Colony formation was evaluated after 7 days. CDV significantly inhibited single cell survival and growth (Fig. 1C) at concentrations that did not inhibit subconfluent cultures, as reflected by the reduced number of colonies and smaller colony size (Fig. 1D) of cells pretreated with 10 or 20 μg/ml CDV. At 50 μg/ml, no colonies could be detected, whereas this concentration still allowed 50% survival in subconfluent cultures (Fig. 1A). Additionally, we determined the effect of CDV on detachment-free survival of F2T-luc2.9 cells seeded on agarose. Again, CDV pretreatment (at 10 and 20 μg/ml) dose-dependently inhibited the clonogenic capacity of the cells (Fig. 1C). These data suggest that CDV may affect the survival of single cells in the blood (in suspension) or after extravasation in the target organ.

### CDV pretreatment inhibits lung metastasis of F2T-luc2.9 cells without affecting primary tumor growth

Prompted by these results we investigated whether CDV inhibits the metastatic potential of F2T-luc2.9 cells after intravenous injection in *SCID* mice. This experimental metastasis model recapitulates all post-intravasation steps of tumor cell metastasis [35].

**Figure 2:**
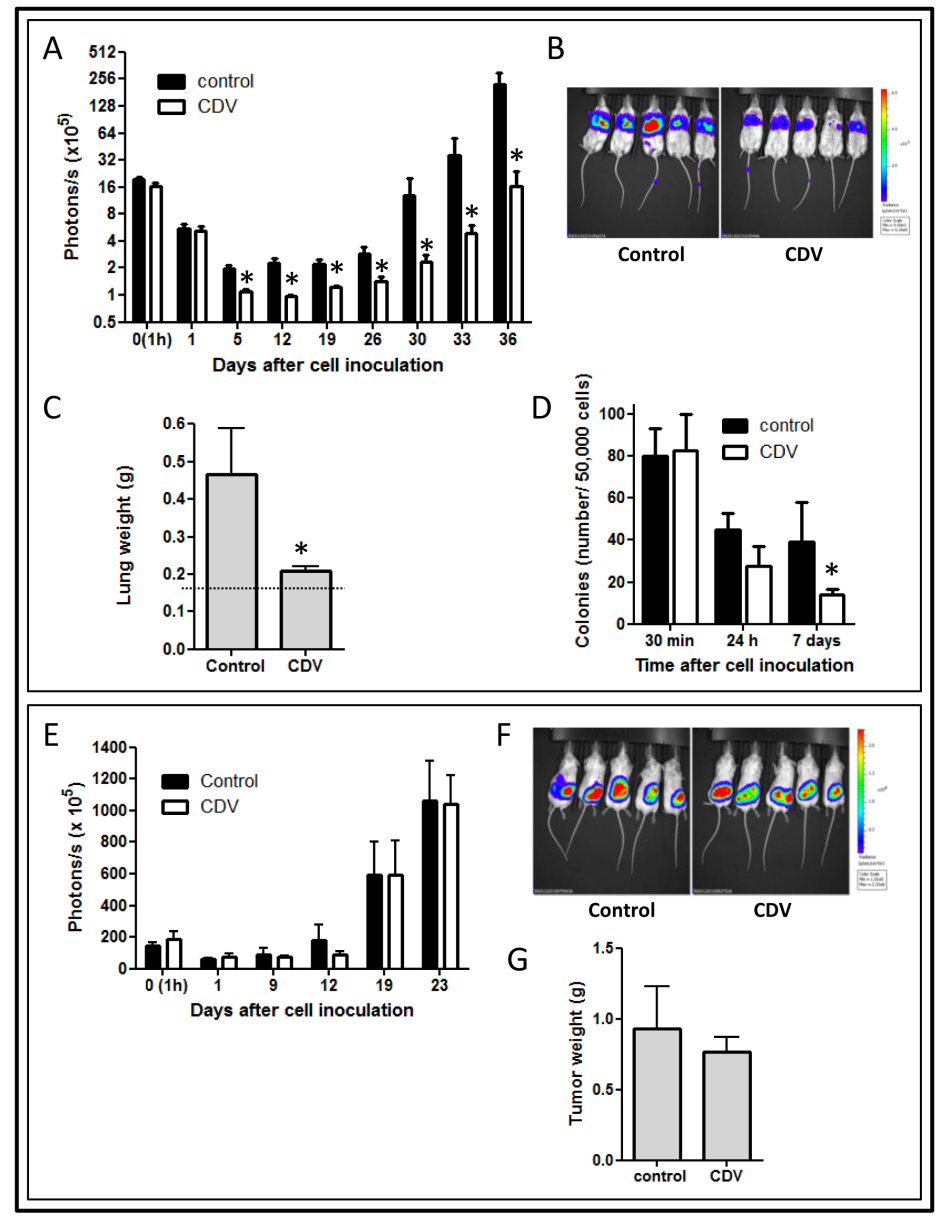
CDV pretreatment of F2T-luc2.9 cells inhibits metastasis but not primary tumor growth F2T-luc2.9 cells were grown for 24 h in the absence or presence of 10 μg/ml of CDV. Next, SCID mice were injected i.v. (A-D) or subcutaneously (E-G) with 10^6^ control or CDV-treated cells. At regular time intervals, the mice were imaged to assess the growth of luciferase-positive metastases (A) or subcutaneous primary tumors (E). At the end of the experiment, the lungs (C) or subcutaneous tumors (G) were dissected and weighed. Representative pictures of bioluminescence are shown (B, F). Dotted line in C indicates normal lung weight. Three independent experiments yielded comparable data. Values are expressed as mean ± S.E.M. of one experiment, n = 5. D, One million cells/200 μl were injected in the tail vein of SCID mice. At indicated time points, lungs were perfused and single-cell suspensions generated. 5 × 10^4^ lung-derived cells were plated into a 10-cm tissue culture dish and cultured in F2T-luc2.9-specific medium. Colonies were counted after 14 days. *p < 0.05; Student-*t*-test.

F2T luc2.9 cells were retained in the lungs within 1h after inoculation into the tail vein (Fig. 2A). The luminescent signal, reflecting the amount of living tumor cells in the lungs, progressively declined during the next days, leaving a small number of viable cells that remained dormant for approximately 3 weeks. Next, disease progression occurred, as indicated by the exponential increase in luminescent signal in the lungs.

In order to evaluate the anti-metastatic properties of CDV, F2T-luc2.9 cells were pretreated with 10 μg/ml of CDV for 24 h, i.e. the lowest concentration that inhibited single cell survival *in vitro* (Fig. 1C). Consistent with the *in vitro* data, CDV pretreatment resulted in a significant reduction in tumor cell survival in the lungs compared with control cells. Indeed, the luminescent signal decreased 8-fold from day 0 to day 5 after injection for control cells *versus* 16-fold for CDV-treated cells (Fig. 2A). Moreover, outgrowth of the surviving cells into macrometastases proceeded more slowly for CDV-treated cells, the luminescent signal increasing more than 100-fold from day 19 to 36 for control cells *versus* 13-fold for CDV-treated cells. This resulted in a significantly reduced weight of harvested lungs at the end of the experiment (Fig. 2C).

CDV was previously shown to inhibit the homing of HPV+ cells to the lungs by inhibition of CXCR4 expression and signaling [34]. However, one hour after cell inoculation equal numbers of luciferase-expressing tumor cells were observed in the lungs of control and CDV-treated groups (Fig. 2A). Also, CDV did not affect F2T-luc2.9 cell adhesion to the extracellular matrix (ECM) components collagen I, laminin, or fibronectin or to an endothelial cell layer (data not shown). Together, these data indicate that CDV does not influence initial retention/homing of F2T-luc2.9 cells in the lungs.

To further investigate the growth potential of lung-arrested tumor cells, lungs were harvested at different time points after cell injection. Then, single cell suspensions were generated and allowed to grow for 2 weeks in hygromycin-containing medium selective for F2T-luc2.9 cells (Fig. 2D). As anticipated, no difference was observed in the number of colonies obtained from lungs containing control or CDV-pretreated cells shortly after injection (30 min). However, the colony-forming capacity of CDV-pretreated cells appeared to be reduced when lungs were harvested 24 h after cell injection; this effect was statistically significant for cells isolated 7 days after cell injection.

On this basis, to assess whether CDV pretreatment affects the intrinsic growth potential of F2T-luc2.9 cells, CDV-pretreated cells (10 μg/ml, 24 h) were injected subcutaneously in *SCID* mice. At variance with the inhibitory effect exerted by CDV pretreatment on the development of F2T-luc2.9 lung metastases, no significant difference in subcutaneous tumor growth could be observed between control and CDV-treated cells (Fig. 2E, F). Accordingly, the weight of the primary subcutaneous tumors harvested 3 weeks after cell injection was not different in the two groups (Fig. 2G). Thus, pretreatment with a low dose of CDV does not affect the intrinsic growth of subcutaneous tumor grafts generated by F2T-luc2.9 cells but hampers single cell survival *in vitro* and metastatic growth *in vivo*.

### Systemic treatment with CDV impairs lung metastasis of F2T-luc2.9 cells

**Figure 3:**
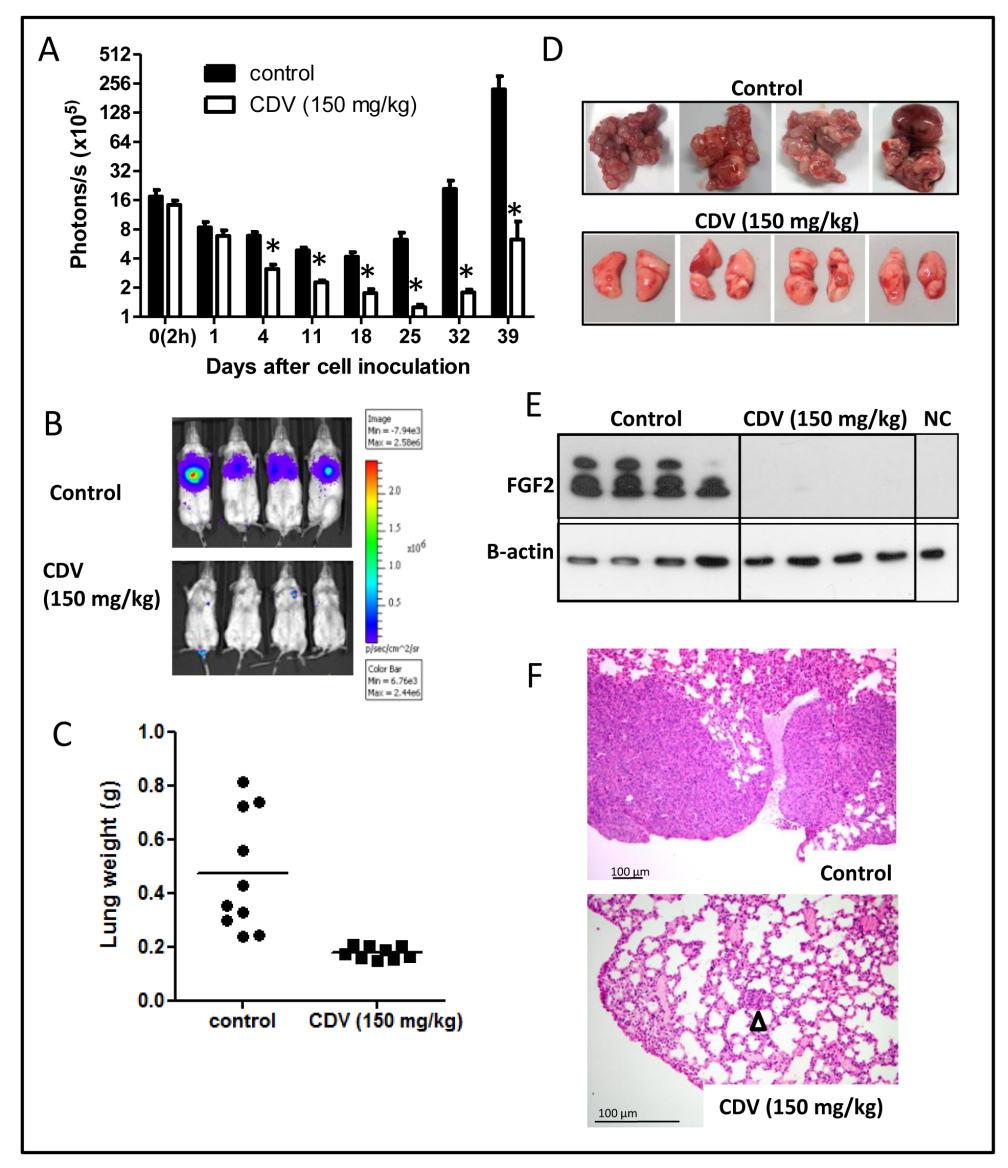
Systemic CDV treatment inhibits the development and growth of F2T-luc2.9 lung metastases A-F, 10^6^ cells/200 μl were injected i.v. in SCID mice. CDV was administered i.p. at 150 mg/kg, once weekly from 2 h after cell inoculation till the end of the experiment. At regular time intervals, the mice were imaged to assess the growth of luciferase-positive metastases (A). At the end of the experiment, the lungs were dissected and weighed (C). Representative pictures of bioluminescence in the lungs of control and CDV-treated mice (B), macroscopic pictures (D) and H&E staining (F) of lungs from control and CDV-treated mice after 39 days are shown. Arrowhead indicates micrometastasis. Two independent experiments yielded comparable data. Results of one experiment are shown. Values are expressed as mean ± S.E.M. (n = 5), *p < 0.05, Student-*t*-test. E, Western blot analysis for FGF2 on homogenized and lysed lungs from control and CDV-treated mice and from normal healthy mice (NC, negative control).

Next, we examined whether CDV also protects against lung metastasis when given systemically after tumor cell injection, i.e. in a therapeutic treatment setting. The anti-metastatic activity of CDV was evaluated at 150 mg/kg i.p., once weekly, a concentration that suppresses FGF2-*T*-MAE primary tumor growth without eliciting systemic toxicity [4]. When treatment was started 1 h after cell inoculation and sustained for 6 weeks, CDV significantly reduced tumor cell survival and outgrowth in the lungs (Fig. 3A), resulting in a markedly reduced metastatic burden and lung weight (470 ± 220 mg *versus* 170 ± 23 mg for control and CVD-treated mice, respectively) (Fig. 3A-C). Macroscopically, lung tumors were detected in all control animals whereas 83% of CDV-treated mice appeared tumor-free (Fig. 3D). Also, Western blot analysis performed on lung homogenates from control and 3F2T.luc2-injected animals harvested 5 weeks after cell injection showed a high expression of FGF2 isoforms, an index of the presence of F2T-luc2.9 cell-derived metastases in control lungs. In contrast, low levels of FGF2 were detectable in the lungs from CDV-treated animals (Fig. 3E). Histological analysis confirmed these observations (Fig. 3F) and showed the presence of microscopically detectable lesions in the lungs of CDV-treated mice, indicating that the development of lung metastases was delayed, but not completely prevented, by systemic CDV treatment.

**Figure 4:**
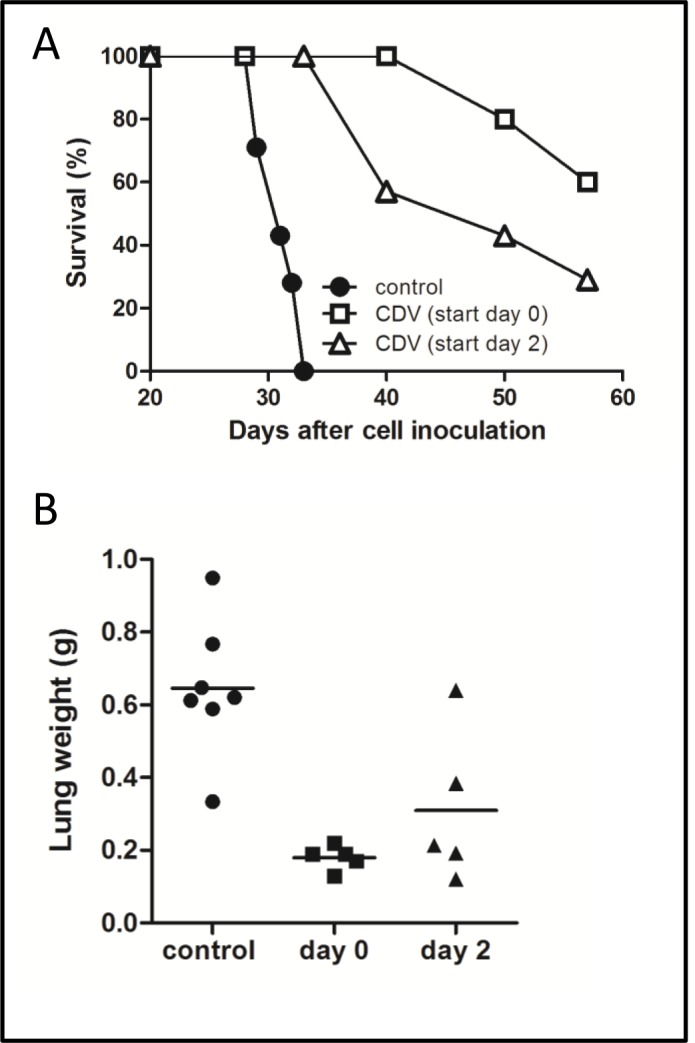
Effect of delayed CDV treatment on metastasis-induced mortality A, B, 2 × 10^6^ F2T-luc2.9 cells were injected into the tail vein of SCID mice. CDV treatment (150 mg/kg i.p., once weekly) was started either 2 h or 2 days after cell inoculation and continued for 5 weeks, when the lungs were dissected and weighed (B). Mortality was determined in a parallel group of animals (A). Two independent experiments yielded comparable data. Results of one experiment are shown.

In a second set of experiments, mice were inoculated with a high amount of cells to assess the effect of systemic CDV treatment on animal survival. As shown in Fig. 4A, control mice died 31 ± 2 days after tumor cell inoculation. In contrast, 60% or 29% of the animals were still alive at day 57 when CDV treatment was initiated 1 h or 2 days after cell inoculation and continued till the end of the experiment. At this time point, surviving animals showed signs of progressing illness and were euthanized. Thus, CDV protects against lung metastasis when treatment is delayed till 2 days post tumor cell injection, but the inhibitory effect was less pronounced compared with the early treatment group (Fig. 4), suggesting that CDV may act at different steps of the metastatic process after tumor cell homing.

### CDV increases nuclear accumulation of p53

CDV has been shown to reduce FGF2-*T*-MAE tumor growth by inducing apoptosis *via* the upregulation of p53 [4,29]. Thus we reasoned that similar mechanisms might be responsible for the anti-metastatic activity of the drug.

**Figure 5:**
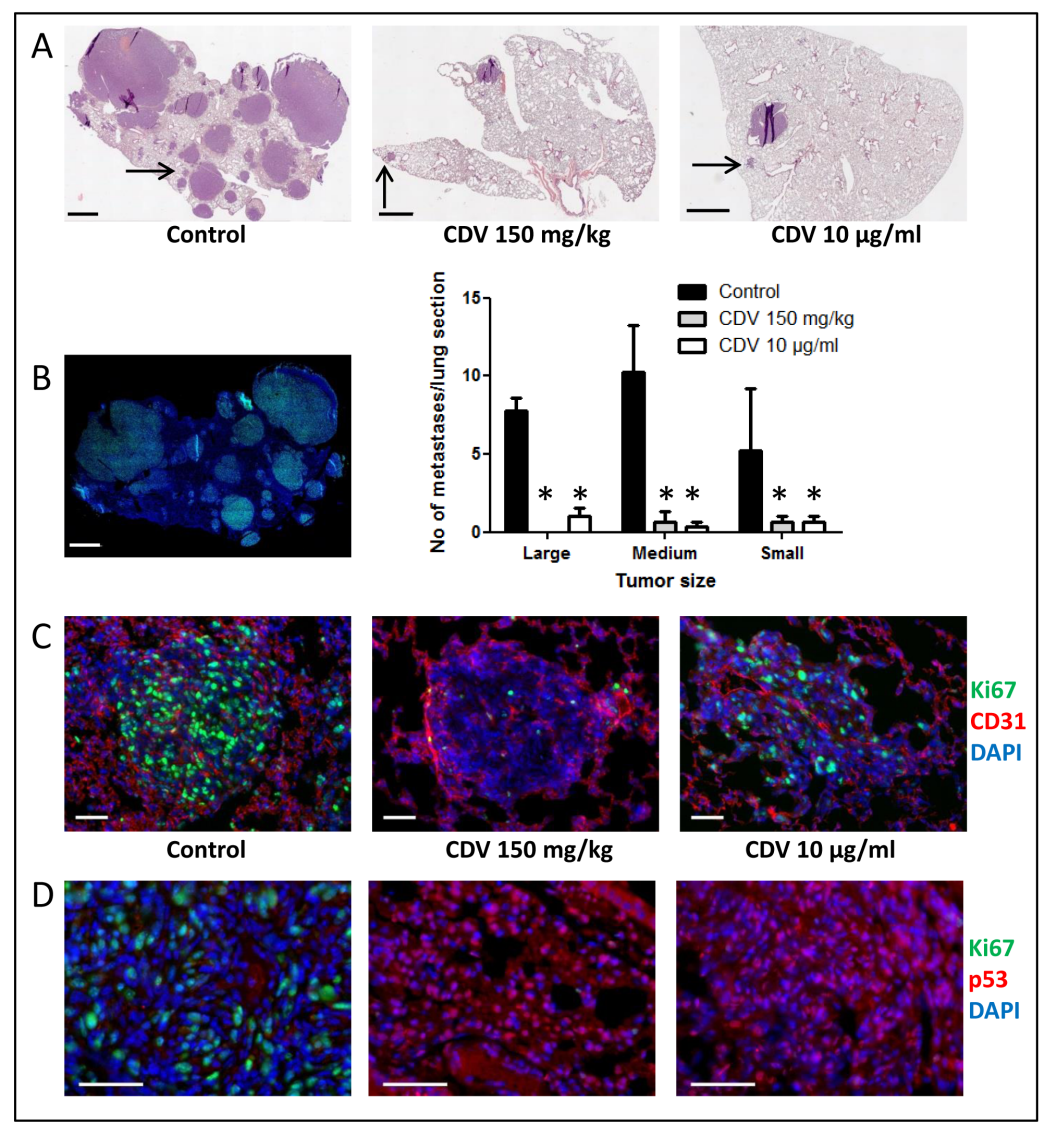
Immunohistochemical analysis of F2T-luc2.9 cell-induced lung metastases Immunohistochemical analysis was performed on lung metastases from mice collected 5 weeks after i.v. injection of either untreated F2T-luc2.9 cells (control), untreated cells with systemic CDV treatment (150 mg/kg.week) or CDV-pretreated (10 μg/ml, 24 h) cells. A, Total lung sections were stained with H&E. Large- (>1 mm), medium- (200 μm-1 mm) and small-size (<200 μm) metastases were counted. Scale bars: 1 mm. Values are expressed as mean ± S.E.M. (n = 5), *p < 0.05, Student-*t*-test. B, Control metastases are highly proliferative as indicated by prominent Ki67 staining. C, Metastases of comparable size (indicated by the arrows in A) were double immunostained with anti-CD31 (red) and anti-Ki67 (green) antibodies, followed by nuclear counterstaining with DAPI (blue). Scale bars: 50 μm. D, Ki67 (green)-negative tumors are detected in CDV groups, but not in control lungs, and are characterized by predominant nuclear p53 staining (red). Scale bars: 50 μm.

Immunohistochemistry (Fig. 5) was performed on lung metastases from mice that were i.v. injected with untreated F2T-luc2.9 cells (control), CDV-pretreated cells or naïve cells followed by systemic CDV treatment. Five weeks after cell injection, metastases of various sizes were detected throughout control lungs (Fig. 5A). Large (> 1 mm) and medium-size (200 μm-1 mm) metastases showed high expression of the proliferation marker Ki67, both in the center and at the tumor rim, and were highly vascularized, whereas micrometastases (<200 μm) stained positive for Ki67 (Fig. 5B, C) but appeared to contain fewer blood vessels or were located perivascularly. The tumor suppressor protein p53 was expressed either in the cytoplasm only (Fig. 5D) or in the cytoplasm and nucleus.

In the group of animals injected with CDV-pretreated cells, few vascularized metastases (1-3 per lung section) were evident. Even though most metastases expressed Ki67, Ki67-negative metastases were characterized by p53 nuclear localization (Fig. 5D). Similarly, metastases in the lungs of CDV-treated mice grafted with naïve F2T-luc2.9 cells were low in number (1-3 per lung section) and small-to medium sized (Fig. 5A). Also in this group, medium-sized lesions did not appear different from control lesions. However, at variance with the other experimental groups, the lungs of CDV-treated mice were characterized by the presence of avascular micrometastases as well as of small lesions that did not express Ki67 (Fig. 5C). Again, p53 was predominantly located in the nucleus of tumor cells in all Ki67-negative lesions in this group (Fig. 5D).

Thus, in keeping with our previous *in vitro* observations on FGF2-*T*-MAE cells [29], CDV may affect the number of F2T-luc2.9 metastases and their growth *in vivo* by reducing Ki67 expression and increasing nuclear accumulation of p53.

### CDV inhibits lung metastasis of B16-F10 melanoma cells in zebrafish and mice

FGF2-dependent B16-F10 melanoma cells represent a prototypic model of experimental metastasis following their injection in the bloodstream of mice [36,37] or zebrafish embryos [38]. On this basis, given the prominent role of FGF2 in tumor progression and metastatic activity of these cells [26 and references therein], we investigated whether the anti-metastatic potential of CDV could be extended also to these virus-independent tumor cells.

**Figure 6:**
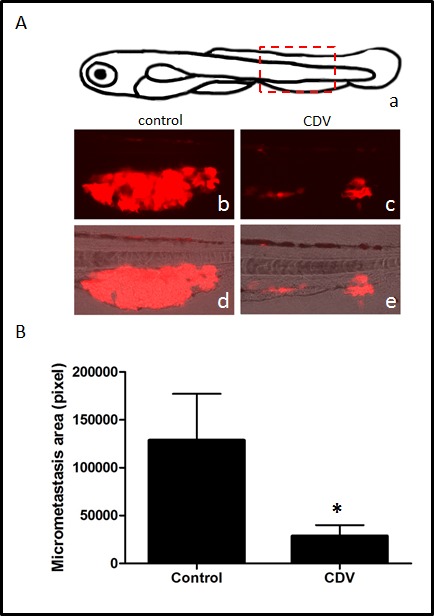
CDV inhibits the metastatic activity of B16-F10 melanoma cells in zebrafish DsRed-B16-F10 cells were injected in the duct of Cuvier of zebrafish embryos at 48 hpf (80-100 cells/embryo). After 24 h, embryos were transferred to fish water in the absence or in the presence of 100 μg/ml of CDV. Micrometastases were observed under a fluorescence stereo microscope and quantified 5 days after cell injection by computerized image analysis of the embryo tails. A) Schematic drawing of zebrafish embryo at 7 days post-fertilization (a): boxed area highlights the tail region photographed at the end of experimentation. Representative micrometastases (red) in control (b,d) and CDV-treated (c,e) animals. B) DsRed-B16-F10 micrometastases were quantified in the whole tail region of each embryo by computerized image analysis. Data are the mean ± S.E.M. of 17-20 embryos per group. *p < 0.05; Student-*t*-test.

In a first set of experiments, DsRed-B16-F10 melanoma cells were injected into the blood circulation of zebrafish embryos at 48 hpf. After 24 h, embryos were transferred in fish water in the absence or in the presence of 100 μg/ml of CDV and the growth of micrometastases in the tail vascular plexus was followed. As shown in Fig. 6, CDV treatment results in a significant reduction of the size of DsRed-labelled micrometastases when assessed 5 days after cell injection. Of note, CDV did not exert any evident toxic effect on the development of zebrafish embryos when compared to vehicle-treated animals (data not shown).

**Figure 7:**
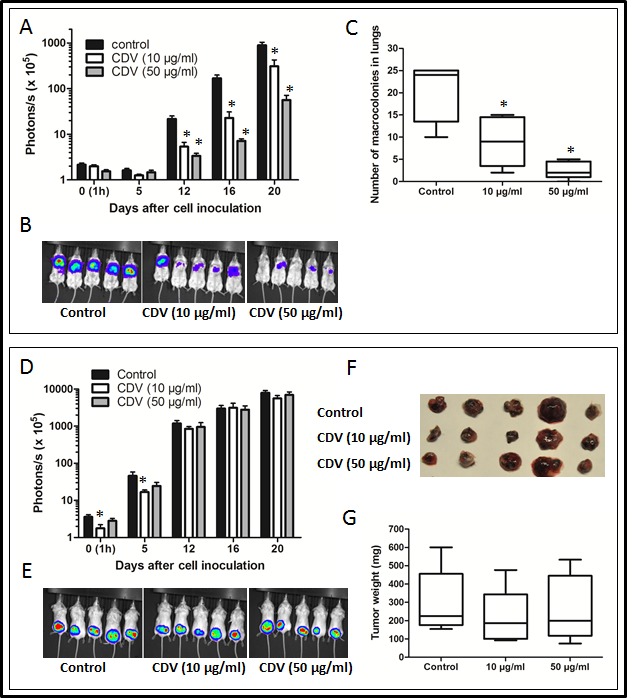
CDV pretreatment inhibits lung metastasis of B16-F10-luc2 melanoma cells B16-F10-luc2 cells were grown for 24 h in the absence or presence of 10 or 50 μg/ml of CDV. Next, SCID mice were injected i.v. (A-C) or subcutaneously (D-G) with 30,000 untreated (control) or CDV-treated cells. At regular time intervals, the mice were imaged to assess the growth of luciferase-positive metastases (A, B) or subcutaneous primary tumors (D, E). At the end of the experiment, the lungs (C) or subcutaneous tumors (F, G) were dissected. The number of macrocolonies in the lungs was determined (C) and subcutaneous tumors were weighed (G). Representative pictures of bioluminescence are shown (B, E). Data are expressed as mean ± S.E.M of 2 independent experiments, n = 10. *p < 0.05; Student-*t*-test.

Next, the effect of CDV on the metastatic activity of B16-F10 melanoma cells was assessed in mice. In accordance with the data obtained with F2T.luc2.9 cells, pretreatment of B16-F10-luc2 cells with CDV abrogated lung metastasis after i.v. injection (Fig. 7A-C) without affecting primary subcutaneous tumor growth (Fig. 7D-G).

## DISCUSSION

Experience with the off-label use of the nucleotide analogue CDV and recent clinical trials indicate that CDV holds great promise as anti-cancer agent for the treatment of high-grade HPV+ carcinomas [16,17]. While CDV preferentially acts on E6/E7 viral oncogene-expressing cells [9], a recent study showed that the compound also inhibits primary glioma growth in human cytomegalovirus (HCMV)-infected as well as in non-infected patients [18], indicating that this compound may exert anti-tumor effects also in virus-independent neoplasms. Indeed, CDV proved effective in the treatment of patients affected by basal cell carcinoma or cutaneous squamous cell carcinoma [19,20]. Moreover, CDV reduces the growth of primary murine B16 melanoma tumor grafts [30] and of Kaposi's sarcoma-like tumors induced by murine FGF2-overexpressing FGF2-*T*-MAE cells [4,27,28].

Thus far, the anti-metastatic activity of CDV had been demonstrated only in one experimental model involving HPV+ cells [34]. Here, we provide novel evidence that CDV inhibits experimental lung metastases induced by virus-independent FGF2-*T*-MAE-derived 3F2T.luc2 tumor cells and B16-F10 melanoma cells, the growth of both cell lines relying on an autocrine activation by FGF2 [4,26].

FGF2 activates FGF receptors (FGFRs) on endothelial cells, resulting in increased tumor vascularization and dissemination of metastatic cells [22]. Accordingly, FGF2 has been shown to play an important role in the pathology of tumors of endothelial cell origin, such as hemangiomas and Kaposi's sarcoma [39-41]. Moreover, levels of circulating FGF2 were suggested to have prognostic value in hematological malignancies, lung cancer, head and neck cancer and melanoma [22,42-46]. In other tumor types, such as gastric and esophageal cancer and pediatric glioma, intratumoral levels of FGF2 were shown to correlate with poor clinical outcome [47-49]. In melanoma, FGF2 upregulation was found to be accompanied by the expression of FGFRs that triggers an autocrine loop of activation required for melanoma growth and survival [22,26,50,51]. Here we show that the injection of DsRed-B16-F10 melanoma cells into the blood circulation of zebrafish embryos results in the growth of micrometastases within 5 days after cell injection. Addition of 100 μg/ml of CDV to the fish water resulted in a significant reduction in the growth of micrometastases, without any evident toxic effect on the development of the embryos. Also, pretreatment of B16-F10-luc2 cells with CDV abrogated single cell survival *in vitro* (data not shown) and lung metastasis after i.v. injection in mice, without affecting primary subcutaneous tumor growth. The anti-metastatic activity of CDV was not limited to B16 melanoma cells. Indeed, our data demonstrate the capacity of the compound to affect also single cell survival of FGF2-dependent 3F2T.luc2 tumor cells and their capacity to induce lung metastases following i.v. injection in immunocompromised animals. These observations pave the way for further studies about the anti-cancer activity of CDV in different FGF2-dependent tumors.

Metastasis is a complex multistep process that involves (i) the detachment of tumor cells from the primary tumor, (ii) trans-endothelial cell migration, (iii) transport of the tumor cells through the blood, (iv) tumor cell arrest in a target organ, (v) extravasation into the tissue and (vi) outgrowth of the secondary tumor [reviewed in 32,33]. Thus, compounds that interfere with tumor cell invasion, detachment-free survival, angiogenesis and resistance to apoptosis may inhibit metastasis. *In vitro* experiments indicate that CDV does not affect F2T-luc2.9 cell migration or adhesion to the ECM components collagen I, laminin, or fibronectin or to an endothelial cell layer (data not shown). Amine [34] demonstrated that pretreatment of HPV16+ cells with CDV reduces tumor cell metastasis by inhibition of *E6* and *E7* viral oncogene expression, leading to decreased CXCR4 levels and reduced homing of the cells to the lungs after i.v. injection. However, we found that CXCR4 protein expression at the cell surface was nearly undetectable both in control and CDV-treated F2T-luc2.9 cells, ruling out the possibility that the anti-metastatic action exerted by CDV on these cells may depend on CXCR4 activity. Accordingly, our *in vivo* observations clearly indicate that CDV does not interfere with F2T-luc2.9 cell homing to the lungs.

CDV reduces the *in vitro* survival of single F2T-luc2.9 cells both when attached to the substratum or in suspension. This occurred at compound concentrations that did not affect the survival of subconfluent cell cultures. Moreover, pretreatment of F2T-luc2.9 cells with CDV reduced lung metastasis after i.v. injection but did not affect the growth of a subcutaneous primary tumor, pointing to a specific effect of CDV at the single cell level. It is well established that the majority of tumor cells die during the first days after arrest in the target organ [52,53]. We found that the massive tumor cell death that occurs in the lungs from day 0 till day 7 after i.v. injection was even more pronounced when F2T-luc2.9 cells were pretreated with CDV and correlated with the reduced colony-forming capacity of these cells when recovered from the lungs 7 days after injection. Together, these data suggest that CDV may affect the survival of single tumor cell colonies or micrometastases in the lung microenvironment [54]. However, despite the pro-apoptotic properties of CDV [4,5,29], we failed to detect apoptotic tumor cells in the lungs of F2T-luc2.9 cell-injected animals 3 and 7 days after grafting (data not shown). This could be due to the paucity of tumor cells present in the lungs at these time points or the fact that apoptotic cells had already been phagocytized and cleared.

The progression from a small avascular nodule to an aggressively growing secondary tumor requires the formation of an intratumoral network of blood vessels [32,55]. CDV caused >90% reduction in the number of F2T-luc2.9 cell-derived lung metastases with complete inhibition of large-size metastases, suggesting that the compound inhibits both the survival of micrometastases as well as macrometastasis outgrowth. Moreover, whereas control metastases were all well vascularized, avascular micrometastases were detectable in the lungs of CDV-treated mice. Accordingly, CDV was shown to reduce the expression of pro-angiogenic FGF2 in F2T-luc2.9 cells *in vitro* [29]. In addition, weekly CDV treatment, that results in a nearly continuous exposure to the drug due to its long half-life [56], was able to maintain micrometastases in a dormant state, as shown by the absence of Ki67 expression in (some of) these lesions. Notably, all Ki67-negative lesions showed predominant nuclear staining of p53, whereas p53 was localized solely in the cytoplasm or in the cytoplasm and nucleus in Ki67-positive metastases. Increased nuclear p53 is likely to result in cell cycle arrest [57], as indicated by the absence of Ki67 staining in these lesions. These data suggest that the anti-tumor/anti-metastatic activity of CDV might be limited to tumors in which downstream p53 signaling is unaffected.

The p53 pathway has been identified as one of the main targets for CDV also in HPV+ tumors in which increased p53 levels are linked to reduced expression of the viral oncogene E6 [9,10]. Further studies will be required to identify the molecular mechanisms linking CDV treatment to increased p53 activity in virus-independent tumors.

Tumor metastases remain the main cause of death in cancer patients, mainly due to late diagnosis and the lack of efficient anti-metastatic drugs. The present study shows for the first time that the FDA-approved antiviral agent CDV inhibits lung metastasis of virus-independent FGF2-driven tumor cells. The concept of using existing drugs for novel applications is very attractive given that treatment can be developed fast and with relatively low costs [58-61]. Developing a new drug can take 10 years or more and costs in excess of 1 billion dollars. Moreover, despite increasing efforts and money being put into research, the number of new drugs approved remains constant. Repurposing old compounds has proven successful in the past and is gaining in popularity [62-65]. In particular, AZT (azidothymidine), a failed anticancer drug, was the first antiviral approved for HIV/AIDS in 1987 [66]. Conversely, the nucleoside analogue Gemzar® (gemcitabine) originally developed as an antiviral agent is now being used to treat pancreatic cancer [67]. Our findings suggest that CDV may have therapeutic potential as an anti-metastatic agent and warrant further studies to select those tumor types that are most likely to benefit from CDV therapy.

## METHODS

### Materials

Cidofovir [(CDV, Vistide^®^] was obtained from Gilead Sciences (Foster City, CA)

### Cell cultures

FGF2-*T*-MAE cells are murine endothelial cells that express high levels of the *M*_r_ 18,000, *M*_r_ 22,000 and *M*_r_ 24,000 molecular weight isoforms of FGF2 [27]. These cells were maintained in Dulbecco's modified minimum essential medium (DMEM, Life Technologies, Inc., Rockville, MD) supplemented with 10 mM Hepes (Life Technologies), 10% fetal bovine serum (FBS), 1 mM sodium pyruvate (Life Technologies) and 500 μg/ml geneticin (Invitrogen). F2T-luc2.9 cells were engineered by selecting the brightest clone of FGF2-*T*-MAE cells transfected with enhanced firefly luciferase (Fluc, Promega, Leiden, The Netherlands) under hygromycin growth conditions (Invitrogen), as described [68]. B16-F10-luc2 murine melanoma cells expressing Fluc (Perkin Elmer, Zaventem, Belgium) were maintained in DMEM, supplemented with 10% FBS.

### Cytotoxicity

Cytotoxicity of CDV was evaluated by the MTS method. Briefly, cells were seeded in 96-well plates at 10,000 cells per cm^2^. After overnight incubation at 37°C, CDV (10, 20, 50 or 100 μg/ml) was added for 24 h. Next, the cells were washed and incubated with fresh medium containing 10% FBS for another 24, 48 or 120 h. To determine the 50% cytotoxic concentration (CC_50_), the medium was removed and 90 μl medium plus 10 μl MTS/PMS (Promega) were added to each well. After an incubation period of 2 h at 37 °C, the optical density of each well was determined at 498 nm in a microplate reader.

### Anchorage-independent growth

F2T-luc2.9 cells were grown in culture flasks and pretreated with different concentrations of CDV (50, 20 or 10 μg/ml) for 24 h. Next, the cells were trypsinized and counted. 5.10^4^ cells were suspended in 2 ml of medium containing 0.3% agar and applied onto 2 ml presolidified 0.6% agar in 6-well plates in the absence of CDV. Medium was replaced twice weekly. After 2 weeks of incubation, cell colonies were stained with 0.1% crystal violet in methanol and counted.

### Single cell survival

F2T-luc2.9 cells were treated and seeded as described above. Next, 300 cells were seeded in 6-well plates in the absence of CDV. After 7 days cell colonies were stained and counted.

### Animals

Female severe combined immunodeficient (SCID) mice, weighing about 20 g were used for all experiments. The animals were bred at the animal facility of the Rega Institute. All studies were done in compliance with the ethical guidelines for animal welfare of the KU Leuven (P028/2011).

### Metastasis assays

Intravenous injection of tumor cells has been used as an experimental model to investigate tumor cell metastasis in mice [35]. Thus, 10^6^ (2.10^6^ for mortality experiments) F2T-luc2.9 cells were injected into the tail vein of SCID mice. Intraperitoneal (i.p.) treatment with CDV (150 mg/kg) was started 1 h after cell injection and continued once weekly. The same volume of phosphate-buffered saline (PBS) was given to control mice. Alternatively, F2T-luc2.9 cells were pretreated with 10 μg/ml of CDV for 24 h. Next, 10^6^ cells (untreated or pretreated with CDV) were injected into the tail vein of SCID mice.

B16-F10-luc2 cells were pretreated with 10 or 50 μg/ml of CDV for 24 h. Next, 30,000 cells were injected into the tail vein of SCID mice.

The mice were imaged at regular time intervals to assess the growth of the luciferase-positive tumor cells. Before imaging, the mice were injected subcutaneously (s.c.) with 200 μl of a 15 mg/ml luciferin solution in PBS. Images were captured by the IVIS spectrum imaging system (Caliper Life Sciences, Hopkinton, MA, USA) and analyzed using the LivingImage software (Caliper Life Sc). At the end of the experiment (as indicated in Results), the lungs were weighed and processed for further analysis.

### Clonogenic assay for viability of lung-arrested tumor cells

10^6^ F2T-luc2.9 cells (treated or untreated with CDV) were injected i.v. in SCID mice. At different time points after cell injection (30 min, 24 h, 7 days) lungs were removed, minced and incubated in DMEM containing 0.25% collagenase type 4 (Sigma-Aldrich) and 300 units DNase I for 1 h at 37°C with agitation. The cells were then passed through a 40 μm cell strainer to collect a single-cell suspension. A total of 5 × 10^4^ lung-derived cells were plated into a 10-cm tissue culture dish. Colonies were stained and counted 14 days later.

### Primary tumor growth in mice

SCID mice were inoculated s.c. with 200 μl of serum-free DMEM containing 10^6^ F2T.luc2.9 cells (untreated or pretreated with 10 μg/ml CDV) or 30,000 B16-F10-luc2 cells (untreated or pretreated with 10 or 50 μg/ml CDV). The mice were imaged at regular time intervals to assess the growth of the luciferase-positive tumor cells. At the end of the experiment, the tumors were excised and weighed.

### Histological and immunohistochemical analyses

At indicated time points, mice were euthanized by i.p. injection of pentobarbital. Lungs were washed with PBS via intracardiac perfusion and fixed intratracheally with 4% paraformaldehyde. Dissected lungs were fixed overnight at room temperature and stored in PBS until paraffin sectioning. Paraffin-embedded sections were deparaffinized in xylene and rehydrated through a decreasing concentration of alcohol. Four micron sections were prepared and subsequently stained with hematoxylin and eosin (H&E). The presence of metastatic nodules in the lungs was evaluated microscopically.

For immunohistochemistry, heat-induced antigen retrieval was done in citrate buffer (pH 6) using a microwave. Sections were permeabilized for 30 min at room temperature with 0.25% Triton X-100 (Sigma) in PBS containing 0.5% BSA, washed with PBS and blocked with 5% goat serum (Sigma) in PBS containing 0.5% BSA for 30 min at room temperature. After washing, sections were incubated overnight at 4°C with a rabbit anti-CD31 (5 μg/ml, abcam), rat anti-Ki67 (1 μg/ml, eBioscience) or mouse anti-p53 (4 μg/ml, Novus Biologicals, Cambridge, UK). After washing with PBS containing 0.01% Tween20, sections were incubated for 3 h at room temperature with an Alexa Fluor 568 goat anti-rabbit antibody (Molecular probes), Alexa Fluor 647 goat anti-mouse antibody (Molecular probes) and Dylight 650 conjugated goat anti-rat antibody (Thermofisher). Sections were washed with PBS containing 0.01% Tween20 and incubated with 2 μg/ml Hoechst (Sigma) for 30 min at room temperature. After washing, sections were mounted with 1% n-propyl gallate and sealed with nail polish. Fluorescent microscopic analysis was done with an Axiovert 200 M inverted microscope (Zeiss, Göttingen, Germany), using a Plan-Apochromat 20x/0.8 objective. Pictures were taken with an AxioCam MRm camera and processed with AxioVision Release 4.6 software (Zeiss).

### Western blot analysis of lung samples

Homogenized lungs were lysed on ice in 500 μl lysis buffer [20 mM Tris-HCl (pH 7.4), 137 mM NaCl, 2 mM EDTA (pH 7.4), 1% Triton X-100, 10% glycerol, 1 mM sodium vanadate, 2 mM sodium pyrophosphate, 1 mM phenylmethylsulfonyl fluoride, 25 mM glycerophosphate and 10 μg/ml leupeptin]. Lysates were cleared by centrifugation and the protein concentration was determined. SDS-PAGE gel electrophoresis of the cell lysates were performed as described [29]. After electrophoresis, proteins were transferred to pretreated Hybond-P polyvinylidene difluoride (PVDF) membranes (Amersham Biosciences). The membranes were incubated for 1h at room temperature in blocking buffer (5 % non-fat dry milk in PBS containing 0.1% Tween) and subsequently for 12 h at 4°C in blocking buffer with primary antibodies raised against β-actin (1/5000, Sigma) or FGF2 (clone FB-8, 1/5000, Sigma). After washing, the membranes were incubated with the corresponding horseradish peroxidase-conjugated secondary antibody (anti-mouse: 1/2000 or anti-rabbit: 1/4000, Dako) in blocking buffer for 25 min at room temperature. Next, the membranes were washed extensively. Immunoreactive proteins were detected by chemiluminescence (ECLplus, Bio-Rad). Samples were collected from 3 independent experiments.

### Metastasis assay in zebrafish embryos

The wild-type AB zebrafish line was maintained at the Zebrafish Facilities of the University of Brescia as described [69]. B16-F10 melanoma cells stably transfected with DsRed fluorescent protein (DsRed-B16-F10 cells) were injected in the blood circulation in the ventral region of the duct of Cuvier of zebrafish embryos (80-100 cells/embryo) at 48 h post-fertilization (hpf). After 24 h, embryos were transferred in fish water in the absence or in the presence of 100 μg/ml of CDV and the growth of tail micrometastases was followed under a fluorescence stereo microscope and quantified 5 days after cell injection by computerized image analysis of the embryo tails as described [38]. After cell injection, embryos were maintained at 33°C throughout the whole experimental period.

### Statistical analyses

Two-tailed Student's *t* test was used to determine the statistical significance of the data; p values < 0.05 were considered significant.
